# Variations of MRI-assessed peristaltic motions during radiation therapy

**DOI:** 10.1371/journal.pone.0205917

**Published:** 2018-10-25

**Authors:** Farshad Mostafaei, An Tai, Eenas Omari, Yingqiu Song, James Christian, Eric Paulson, William Hall, Beth Erickson, X. Allen Li

**Affiliations:** 1 Department of Radiation Oncology, Medical College of Wisconsin, Milwaukee, Wisconsin, United States of America; 2 Union Hospital Cancer Center, Huazhong University of Science and Technology, Wuhan, China; North Shore Long Island Jewish Health System, UNITED STATES

## Abstract

**Purpose:**

Understanding complex abdominal organ motion is essential for motion management in radiation therapy (RT) of abdominal tumors. This study investigates abdominal motion induced by respiration and peristalsis, during various time durations relevant to RT, using various CT and MRI techniques acquired under free breathing (FB) and breath hold (BH).

**Methods:**

A series of CT and MRI images acquired with various techniques under free breathing and/or breath hold from 37 randomly-selected pancreatic or liver cancer patients were analyzed to assess the motion in various time frames. These data include FB 4DCT from 15 patients (for motion in time duration of 5 sec), FB 2D cine-MRI from 4 patients (time duration of 1.7 min, 1 second acquisition time per slice), FB cine-MRI acquired using MR-Linac from 6 patients in various fractions (acquisition time is less than 0.6 seconds per slice), FB 4DMRI from 2 patients (time duration of 2 min), respiration-gated T_2_ with gating at the end expiration (time duration of 3–5 min), and BH T_1_ with multiphase dynamic contrast in acquisition times of 17 seconds for each of five phases (pre-contrast, arterial, venous, portal venous and delayed post-contrast) from 10 patients. Motions of various organs including gallbladder (GB) and liver were measured based on these MRI data. The GB motion includes both respiration and peristalsis, while liver motion is primarily respiration. By subtracting liver motion (respiration) from GB motion (respiration and peristalsis), the peristaltic motion, along with small residual motion, was obtained.

**Results:**

From cine-MRI, the residual motion beyond the respiratory motion was found to be up to 0.6 cm in superior-inferior (SI) and 0.55 cm in anterior-posterior (AP) directions. From 2D cine-MRI acquired by the MR-Linac, different peristaltic motions were found from different fractions for each patient. The peristaltic motion was found to vary between 0.3–1 cm. From BH T_1_ phase images, the average motions that were primarily due to peristalsis movements were found to be 1.2 cm in SI, 0.7 cm in AP, and 0.9 cm in left-right (LR) directions. The average motions assessed from 4DCT were 1.0 cm in SI and 0.3 cm in AP directions, which were generally smaller than the motions assessed from cine-MRI, i.e., 1.8 cm in SI and 0.6 cm in AP directions, for the same patients. However, average motions from 4DMRI, which are coming from respiratory were measured to be 1.5, 0.5, and 0.4 cm in SI, AP, and LR directions, respectively.

**Conclusion:**

The abdominal motion due to peristalsis can be similar in magnitude to respiratory motion as assessed. These motions can be irregular and persistent throughout the imaging and RT delivery procedures, and should be considered together with respiratory motion during RT for abdominal tumors.

## Introduction

Inter and intra-fractional organ motions resulting from respiratory motion can be more than 2 cm and have been recognized as a primary challenge in the delivery of radiation therapy (RT) for tumors in the thorax and abdomen [1–7]. These motions are patient specific and can vary between and within fractions, in either or both amplitude and frequency [8, 9]. Also, motion phase can vary from one anatomical location to another [10–13]. A common method to account for these motions is to add a large margin (e.g., 1 cm) to the clinical target volume (CTV), ensuring adequate coverage of the CTV. However, large margins increase the dose to nearby normal tissues and can lead to radiation-induced toxicities. Image guided radiation therapy (IGRT) and tumor tracking during treatment have been used to reduce these large margins [2].

Organ motion in the abdomen can be quite complex as there may be peristaltic motion in addition to motion induced by respiration as described above. It is essential to assess and manage all of these motions (e.g., respiratory and peristaltic) and their interplay for high precision radiation therapy. Without appropriately accounting for these motions, the target volume could be under-dosed or missed, and nearby organs at risk (OARs) could be overdosed.

Previous studies have investigated organ motion in the abdomen and respiratory motion using different imaging modalities (e.g. 4DCT, cine-MRI, x-ray fluoroscopy and ultrasound) [5, 14–19]. Shimizu *et al*. [20] reported the liver motion in AP and LR directions, as measured by cine-MRI (AP: 8 mm, LR: 9 mm) and 4DCT (AP: 5 ± 3.1 mm, LR: 3 ± 2 mm). Feng *et al*. [14] and Goldstein *et al*. [1] reported the pancreas motion of 8 ± 3 mm using cine-MRI and of 3 ±1.7 mm using 4DCT.

Whereas abdominal organ motion due to breathing is well understood and was investigated by previous studies, little attention has been paid to peristaltic motion. Kumagal *at al*. [20] reported the dose variation to the pancreas and the duodenum due to bowel gas movement. Nakamoto *et al*. [9] showed bowel motion increased the artifacts in PET/CT images due to attenuation differences.

These studies, however, measured overall organ motion in a time frame up to 2 min, which cannot provide full organ motion information for RT. This study aims to systematically investigate overall abdominal organ motions in the time scale (2–45 min) relevant to RT delivery and to investigate the characteristics of non-respiratory motion. Imaging data acquired with various modalities in various time frames were used and analyzed to assess organ motion. In particular, organ motion due to peristaltic motion was assessed using free breathing (FB) and breath hold (BH) imaging in the time frames of seconds to minutes, and the relative motion between the head of pancreas (HP) vs. the duodenum (D) was assessed using 3DMRI.

## Method and materials

Various types of MRI data collected in our routine clinical practice for randomly selected liver or pancreatic cancer patients were analyzed retrospectively to assess the organ motions. All MRI data were acquired using either a 3-Tesla MRI (Siemens Healthcare, Erlangen, Germany) for radiation therapy (RT) simulation, or a 1.5-Tesla MR-linac systems (Elekta Ltd & Philips) for longitudinal imaging study. The simulation data were used to assess motion variations between different patients, while the data from MR-Linac were for motion variation between different fractions for the same patient. All the patients scanned on the MR-Linac signed informed consent before the scanning. The retrospective analysis of the MRI data from MRI simulation were also approved by the Medical College of Wisconsin Institutional Review Board. On these images, the head of pancreas (HP), tail of pancreas (TP), duodenum (D), liver, right kidney (RK), left kidney (LK), gallbladder (GB) and stomach were contoured on these images using MIM software^™^ (Cleveland, OH).

A total of 33 cine MRI sets acquired during RT simulation or select fractions for 10 patients with pancreatic cancer were analyzed. Several fast 2D cine MRI were acquired in sagittal, coronal and axial planes for each patient. Free breathing (FB) cine-MRI acquired during MRI simulation from 4 patients (time duration of 1.7 min, 1 second acquisition time per slice), and FB cine-MRI acquired using MR-Linac from 6 patients in various fractions (acquisition time is less than 0.6 seconds per slice) were analyzed. Motions of various organs including GB and liver were measured based on these cine MRI data. The GB motion includes both respiration and peristalsis, while liver motion is primarily respiration [21]. By subtracting liver motion (respiration) from GB motion (respiration and peristalsis), the peristaltic motion, along with small residual motion, was obtained.

The T_1_-weighted 3DMRIs acquired under BH with acquisition times of 17 seconds were used to estimate non-respiratory motion. Patients practiced BH technique to make sure that BH is tolerable for all of them, and there is no change during the acquisition. The T_2_-weighted 3DMRI data acquired under respiration gating at the end expiration (50% phase), with time durations of 3–5 min based on HASTE (spin-echo) imaging sequence, were also used [22]. For the T_1_ weighted 3DMRI, multiphase dynamic contrast, MRI data was acquired as well as pre and 5 min post gadolinium-based contrast agent injection images. In addition, T_2_ weighted MRI images were acquired ~30 mins prior to contrast. Organ motion was then measured with respect to T_2_ images for all contours in the SI, AP, and LR directions. The acquisition times for which data is analyzed are as follows: t_1_ = 0 minutes (T_2_ 30 mins prior to t_2_), t_2_ = pre contrast injection, t_3_ = 2 mins following t_2_ (arterial phase), t_4_ = 0.5 mins following t_3_ (venous phase), t_5_ = 0.5 mins following t_4_ (portal venous phase), and t_6_ = 2.5 mins following t_5_ (5 min post contrast). All centroid displacements were measured relative to the T_2_ MRI phase. Table 1 shows the inter-sequence delay between ends of T_2_ weighted to start of T_1_ weighted phases used for data analysis.

**Table 1:** The inter-sequence delay (min) at the end of T_2_ weighted for 10 patients with 3DMRI.

	Patient ID	1	2	3	4	5	6	7	8	9	10
	Time Ref										
1	**T2 HASTE**	0	0	0	0	0	0	0	0	0	0
2	**T1 BH PRE**	37.5	42.5	40.6	30.75	31	18.8	42.6	28.5	44.45	46
3	**T1 BH Arterial**	40	45.37	43.87	33.22	34.5	20.73	46	32.35	50.57	49.5
4	**T1 BH Venous**	40.5	45.88	44.5	33.7	35.2	21	46	32	51	50.2
5	**T1 BH Portal Venous**	41	46.4	45.3	34	35.7	21.62	46.4	33.48	51.58	51
6	**T1 BH Post ~5 Minute Delay**	43	51.3	47	-	43	33	-	41	51.18	-

Free breathing 4DMRI data were acquired with a hybrid 3D golden angle radial stack of stars sequence of our own design [23]. The 4DMR images were found to be more flexible in comparison to 4DCT in term of selecting image plane and respiratory phase. The data was sorted into 10 respiratory phases from 0% to 90% (50%: end of expiration, 0%: end of the inspiration). The centroid motion of structures on the 4DMRI images throughout the 10 breathing phases, with respect to the 50% phase, were measured in the SI, AP and LR directions.

To demonstrate the significance of peristaltic motion, various types of CT data, including contrast-enhanced 3DCT, 4DCT, and sequential dual-energy CT were collected per our usual clinic routine for selected patients were analyzed. 4DCT data sets of 15 randomly selected patients with pancreatic and liver cancer acquired during RT simulation were used for this study. The 4DCT data were acquired on a GE LightSpeed four-slice CT scanner (GE Medical Systems) using a RPM system (Varian Inc.) with patients in their treatment immobilization. A sequential 4DCT protocol was used in which the CT scans at a couch location for a time period of about 5 seconds (breathing period plus one second) before it moves to another couch location. The data were sorted into 10 respiratory phases from 0% to 90% (50%: end of expiration, 0%: end of the inspiration). The HP, TP, duodenum, liver and stomach were contoured on all 10 phases using MIM software^™^ (Cleveland, OH). The centroid motions of these structures throughout the 10 breathing phases with respect to the 50% phase were measured in the SI, AP and LR directions.

This study includes three phases:

1. Finding peristaltic motion using cine-MRI (MR-Sim & MR-linac) and compare it with 4DCT.
2. Calculating peristaltic motion using BH 3DMRI
3. Relative motion between pancreatic head vs duodenum using 3DMRI and 4DCT.

## Results

Abdominal organ motion was measured and analyzed using different imaging modalities. Each organ motion was investigated to find the peristaltic motion. The following motion results data are presented in terms of image duration time (seconds to minutes).

**Fig 1:**
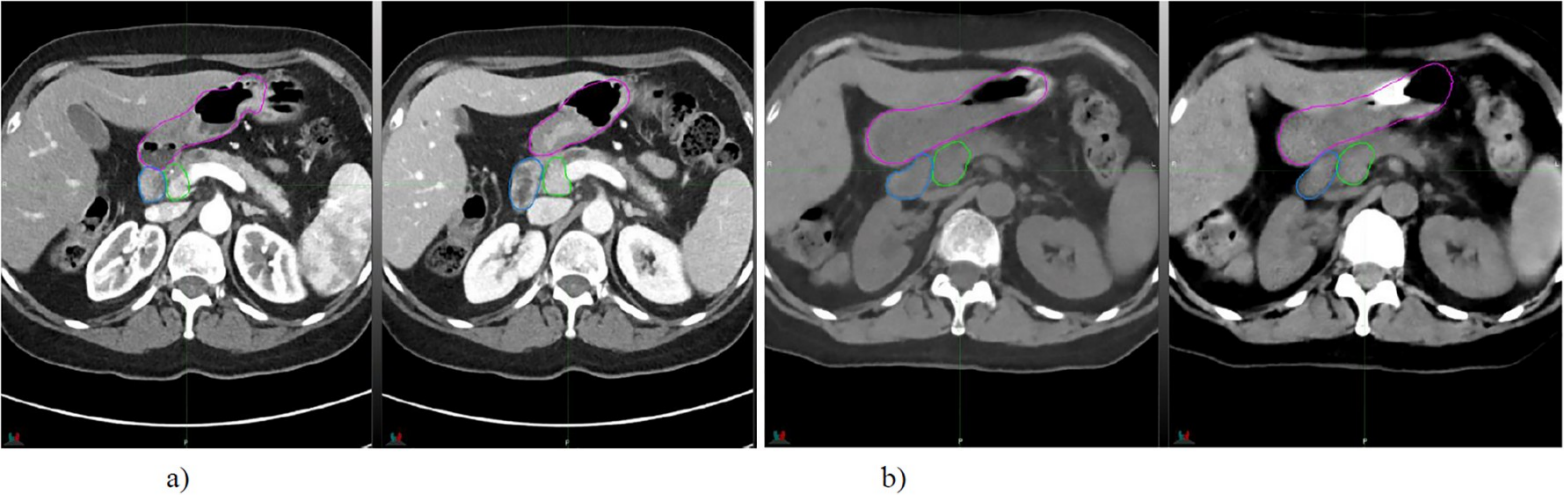
a) two contrast CT images of a patient with one min delay, b) two dual energy CT of the same patient with ~15 seconds delay. Red: stomach, Blue: duodenum and Green: head of pancreas. The shape and direction of the duodenum changed between subsequent minute.

To demonstrate the effect of peristaltic motion, two phases of contrast CT images of a sample patient with one minute delay and the sequential dual energy CT images of the same patient with ~15 seconds delay are shown in Fig 1. It is shown that the duodenum and/or head of pancreas was dislocated and deformed due to gas movement inside the stomach.

**Fig 2:**
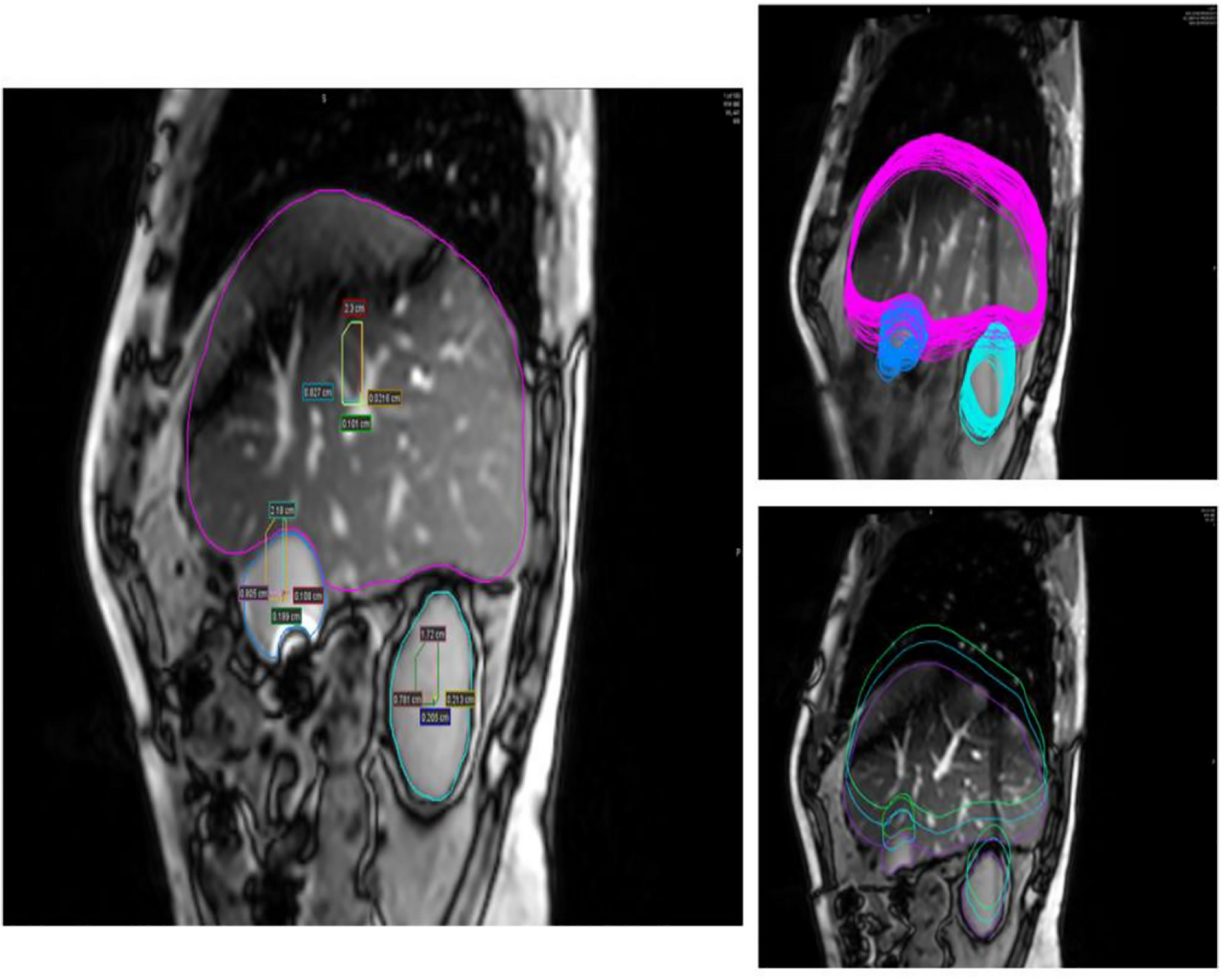
Liver, GB and kidney motions in 100 seconds using 2D cine-MRI in SI and AP directions. Center of mass motions of liver and GB in both directions (left). Maximum and minimum motion of liver and GB due to respiratory (right).

**Fig 3:**
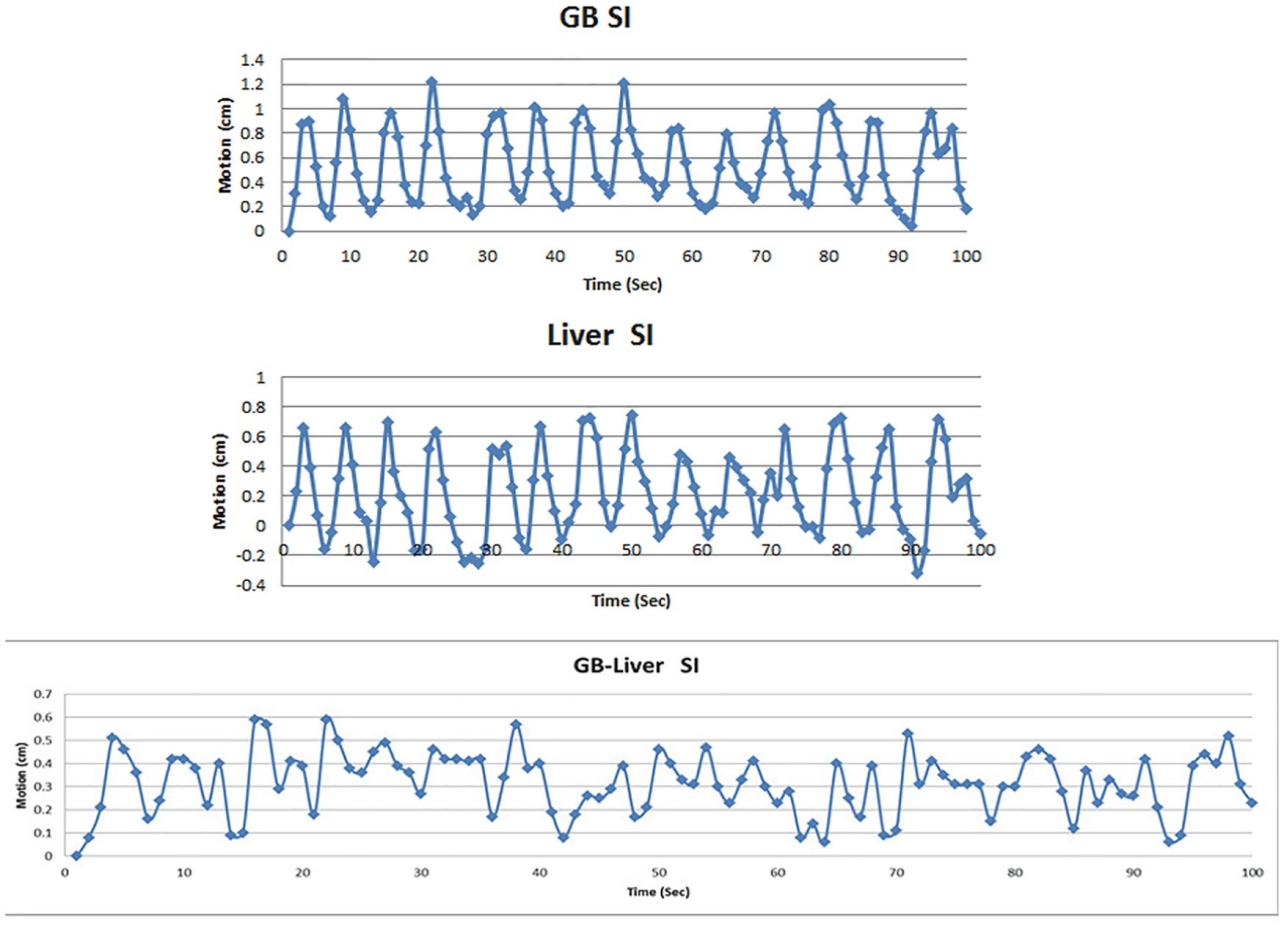
Motion of GB (top) and liver (middle), and subtraction of GB motion from liver motion (bottom) in SI direction from 2D-cine MR.

From 2D cine-MRI acquired during MR simulation, the residual motion beyond the respiratory motion in 1.7 min was found to be up to 0.6 cm in SI and 0.55 cm in AP directions. From 2D cine-MRI acquired by the MR-Linac, different peristaltic motions were found in different fractions for each patient. The peristaltic motion was found to vary between 0.3–1 cm. The peristaltic motion for one patient is shown in Table 2. For each patient the respiratory motion pattern from GB and liver in different fractions were found to be similar, however, after subtraction, the motion pattern and amplitude were found to be different in each fraction. This indicates the non-periodic and non-predictable nature of peristaltic motion although the residual motion that corresponds to the high frequency component of the motion pattern could come from the respiratory deformation of liver and gallbladder. Fig 2 demonstrates liver and GB motions using 2D-cine MR. Subtraction of GB motion from liver motion in the SI direction over 100 seconds is shown in Fig 3.

**Table 2:** The peristaltic motion for one patient.

		2D Cine-MRI (FB) Acquired by MR-Linac	2D Cine-MRI (FB) Acquired by MR-SIM
Acquisition Time per frame		0.6 Seconds	1 Seconds
**First fraction**	SI	Liver:1 cm GB:1.1 cm Peristalsis:0.3 cm	Liver:1.3 cm GB:1.2 cm Peristalsis:0.6 cm
**Second fraction**	SI	Liver:1.3 cm GB:1.6 cm Peristalsis:0.4 cm	
**Third fraction**	SI	Liver:0.8 cm GB:1 cm Peristalsis:1 cm	
**Fourth fraction**	SI	Liver:1.8 cm GB:1.4 cm Peristalsis:0.6 cm	

The motion of abdominal organs due to peristalsis is substantial (up to 1 cm) and the pattern of the motion is complex compared to the respiratory motion based on 2D cine MRI. The motion can vary day to day for a given patient and can be irregular and persistent throughout RT imaging and delivery.

Cine-MRI and 4DCT images of two representative patients were also compared (Table 3). In comparison to 4DCT, cine-MRIs detected larger differences in hepatic tumor motion. This is most notable in the SI direction. The average motion for abdominal organs as assessed from 4DCT, with respect to the 50% phase (end of expiration) were 1 cm in SI and 0.3 cm in AP directions, which were generally smaller than the motion assessed from cine-MRI, 1.8 cm in SI and 0.6 cm in AP directions, for the same patients.

**Table 3:** FB motions from 2 patients with 4DCT and cine-MRI.

		4DCT (FB)	2D Cine-MRI (FB)
Acquisition Time		5 Seconds	1.7 Minutes
Patient # 1	SI	Liver:1.1 cm GB:1.5 cm	Liver:2.3 cm GB:2.2 cm
	AP	Liver:0.2 cm GB:0.5 cm	Liver: 0.8 cm GB:0.8 cm
Patient # 2	SI	Liver:0.85 cm GB:0.3 cm	Liver:1.2cm GB:0.96 cm
	AP	Liver:0.3 cm GB:0.2 cm	Liver:0.4 cm GB:0.4 c

**Fig 4:**
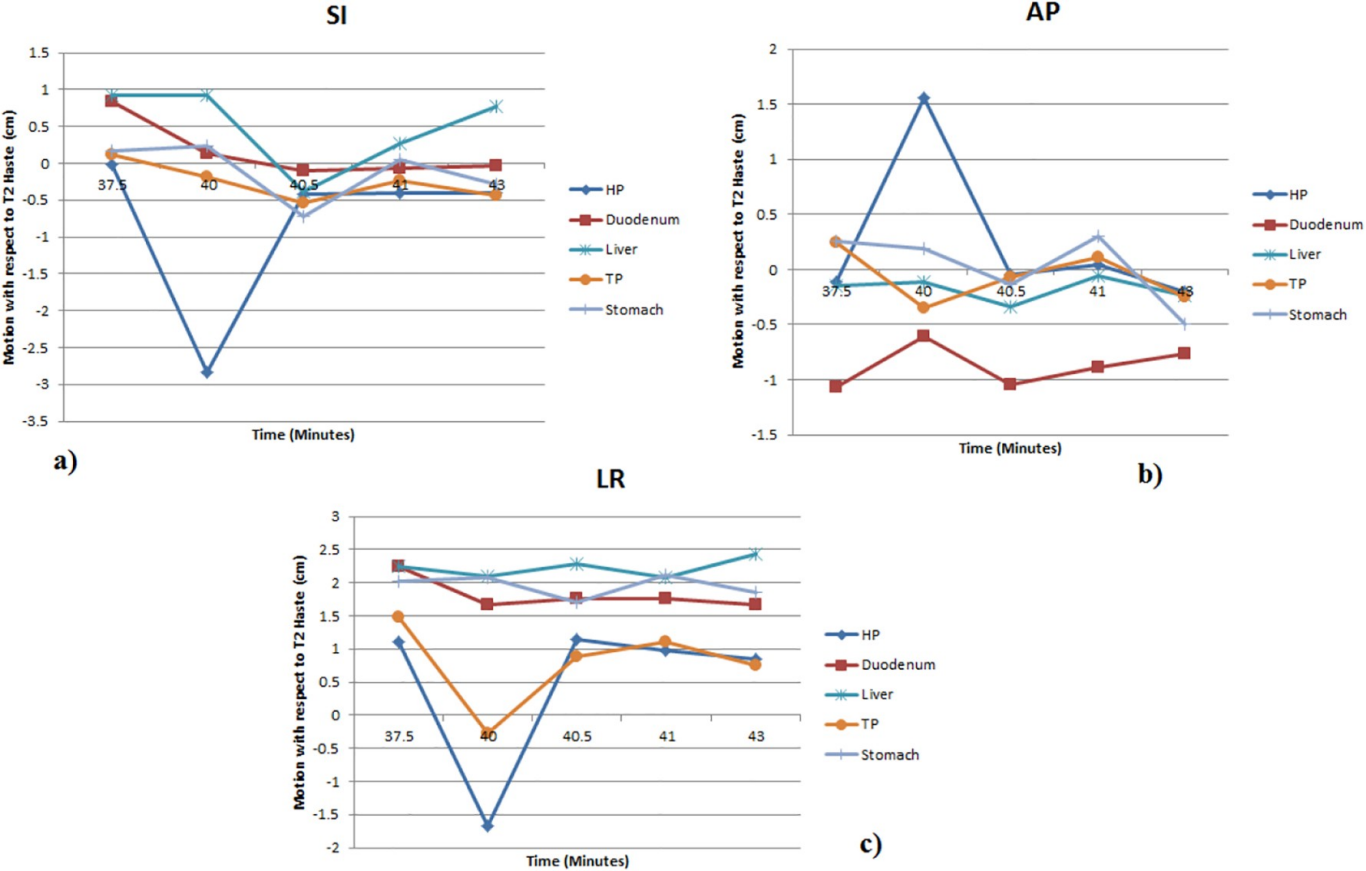
The abdominal organ motion for one patient in the superior-inferior (SI), anterior-posterior (AP), and left-right (LR) directions from all T_1_ phases with respect to T_2_ weighted. HP: head of pancreas, TP: tail of pancreas, duodenum, liver, and stomach.

In order to investigate motion related to peristalsis, the abdominal organ motion in the time range of 2.5–45 minutes were analyzed using 3DMRI. The abdominal organ motion in SI, AP, and LR directions from all T_1_ phases with respect to T_2_ from one patient are shown in Fig 4. Table 4 shows the average and range of abdominal organ motions for 10 patients in different directions.

**Table 4:** Average and range of all 10 patient’s organ motions in different directions from 3DMRI (mean ± standard deviation in cm).

Organ	HP	TP	Liver	Duodenum	GB	Stomach
Average all phases for 10 Patients (cm)						
SI	0.9 ± 0.3 (range:0.02–3.7)	0.7 ± 0.3 (range:0.05–4.3)	0.8 ± 0.3 (range:0.04–3.9)	1.1 ± 0.4 (range:0.1–5.4)	0.9 ± 0.4 (range:0–4.4)	1.2 ± 0.3 (range:0.04–4)
AP	0.5 ± 0.2 (range:0–1.8)	0.3± 0.1 (range:0.02–1.8)	0.3 ± 0.1 (range:0.03–1.3)	0.7 ± 0.1 (range:0.2–1.2)	0.5 ± 0.1 (range:0.03–1.6)	0.5 ± 0.1 (range:0.01–1.3)
LR	0.4 ± 0.2 (range:0.03–1.7)	0.4 ± 0.1 (range:0.04–1.5)	0.5 ± 0.2 (range:0.05–2.4)	0.9 ± 0.2 (range:0.2–2.2)	0.4 ± 0.1 (range:0.04–1.7)	0.7 ± 0.2 (range:0.1–2.1)

The average motion of duodenum and pancreatic head for all T_1_ weighted sequences was found up to 1 cm as shown in Table 4. By investigating the range of organ motion, a patient with large duodenum and pancreatic head motion of 3.7 cm and 5.4 cm in the SI direction was found due to peristalsis motion. However, by studying the duodenum and the pancreatic head motion in various T_1_ phases, the motion up to 5 mm for the same patient was found.

### Relative motion between pancreatic head vs duodenum using 3DMRI and 4DCT

The relative motion between the head of the pancreas and duodenum was measured in all phases. The motion data between different phases in all three directions showed no correlation between the motions of these two structures. In some cases, the pancreatic head and duodenum moved along a different direction for the same phase. For example, a duodenal displacement of 3 mm superiorly and head of the pancreas displacement of 5 mm inferiorly was observed. In other cases, while little motion of the pancreas was measured, a large duodenal displacement was found. For example, a pancreas displacement of less than 1 mm in the superior direction was observed while the displacement of the duodenum varied and reached a maximum of 10 mm. If a phase in which the maximum separation between OARs and the target (pancreatic head) can be determined, radiation treatment can be designed to reduce normal tissue toxicity.

The relative motion between the head of the pancreas and duodenum was measured in all phases and showed the motion as large as 1.85 cm in left-right direction. This large motion between OARs and the target (pancreatic head) may be used to design adaptive radiation treatment strategy, for reducing normal tissue toxicity.

The detailed peristalsis motion of the pancreatic head and duodenum, as measured from each BH T_1_ phases, are compared in Table 5. Organ motion due to peristalsis was found to be dominant with the BH method.

**Table 5:** The peristalsis motion of the pancreatic head and duodenum, as measured from each BH T_1_ phase (mean ± standard deviation in cm).

3DMRI phases		T_2_ haste	T_1_ Pre-contrast	T_1_ Arterial	T_1_ Venous	T_1_ Portal venous	T_1_ Post-contrast
Average inter-sequence delay (min)			34	37.6	38.1	38.7	44.8
Pancreatic Head (cm)	SI		0.55 ± 0.3	0.8 ± 0.4	0.45 ± 0.3	0.5 ± 0.3	0.8 ± 0.4
	AP		0.3 ± 0.2	0.4 ± 0.2	0.3 ± 0.1	0.2 ± 0.1	0.4 ± 0.2
	LR		0.3 ± 0.1	0.4 ± 0.2	0.3 ± 0.1	0.3 ± 0.1	0.4 ± 0.1
Duodenum (cm)	SI		1 ± 0.5	0.8 ± 0.4	0.9 ± 0.5	0.75± 0.5	1.2 ± 0.5
	AP		0.6 ± 0.1	0.45 ± 0.1	0.6 ± 0.1	0.45± 0.1	0.3 ± 0.1
	LR		0.7 ± 0.2	0.6 ± 0.2	0.7 ± 0.2	0.6 ± 0.2	0.6 ± 0.2
Duodenum relative to Pancreatic Head (cm)	SI	0.4	0.13	0.4	0.14	0.2	0.1
	AP	0.33	0.1	0.31	0.11	0.7	0.4
	LR	1.85	1.7	1.3	1.6	1.5	1.3

The relative motion between the pancreatic head and duodenum using 4DCT was also observed. A duodenal displacement of 8 mm superiorly and pancreatic head displacement of 2 mm inferiorly at the 60% phase was observed for one patient studied. Pancreas displacement of less than 1 mm in the superior direction was observed over 6 phases, at these same phases, the displacement of the duodenum varied and reached a maximum of 10 mm for another patient.

**Fig 5:**
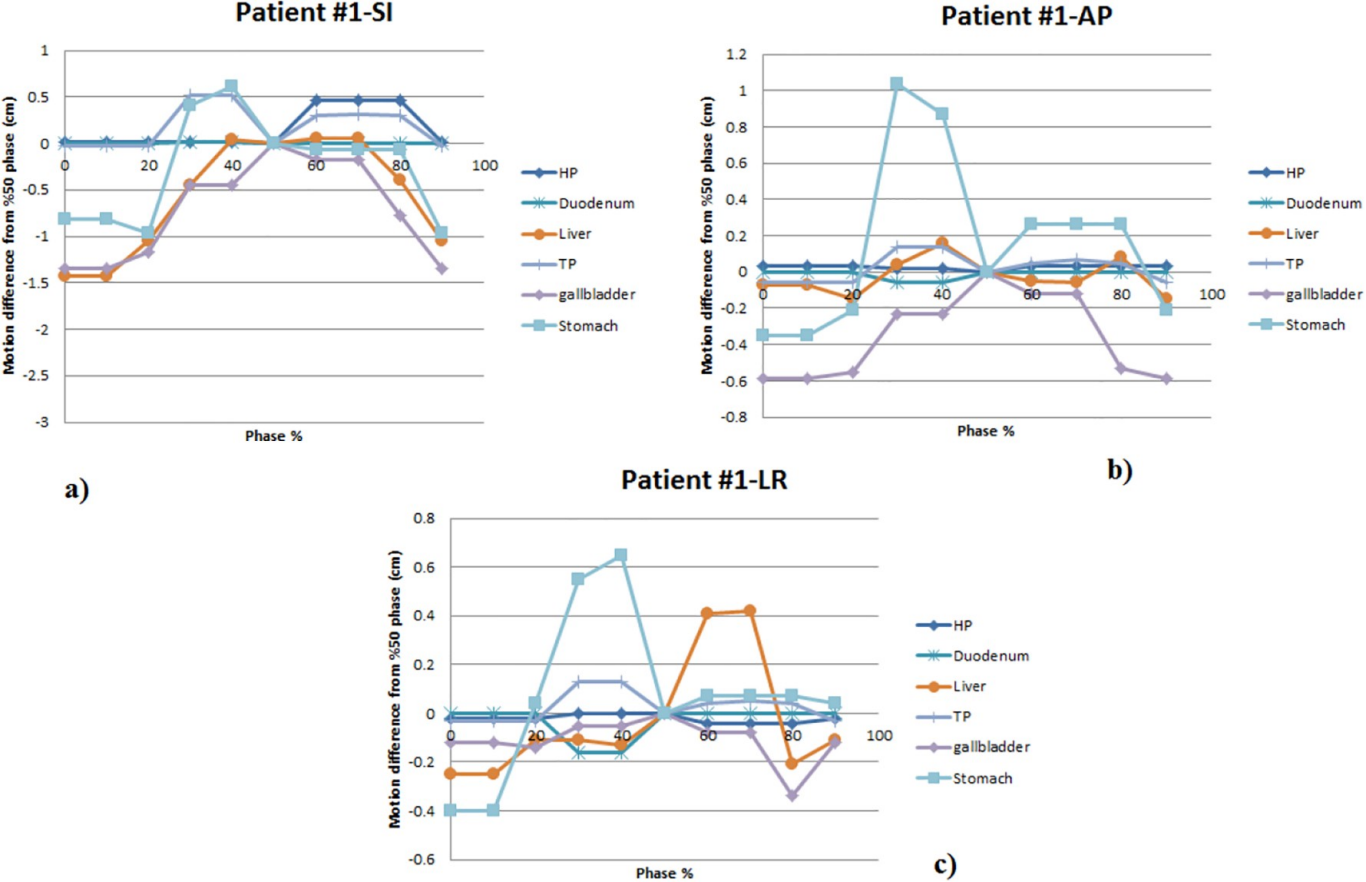
The abdominal motions in the superior-inferior (SI), anterior-posterior (AP), and left-right (LR) directions analyzed from 4DMRI with respect to the 50% breathing phase. HP: head of pancreas, TP: tail of pancreas, duodenum, liver, gallbladder and stomach.

The abdominal motions measured with 4DMRI in the SI, AP, and LR directions from one of the selected patients are shown in Fig 5. The average organ motion from 4DMRI in 2 min was measured to be 1.5 cm, 0.5 cm, and 0.4 cm in SI, AP, and LR directions, respectively. These motions should be the combination of peristalsis and respiration. The stomach motion was also found up to 1.5 cm, 1.4 cm and 1.1 cm in SI, AP, and LR directions respectively.

## Discussion

Internal organ motions relevant to RT should be assessed in the time frames covering imaging acquisition (1–3 min for CT, 5–20 min for PET, 5–45 min for MRI) for RT simulation and delivery guidance as well as delivery (beam on) duration (2–10 min). These motions can be complex in the abdomen as there is peristaltic motion in addition to respiratory motion. In this work, by analyzing various types of MRI and CT images acquired at different time scales under either FB or BH, we have measured internal organ motion in the abdomen with an emphasis on peristaltic motion.

4DCT was found to underestimate motions compared to cine-MRI. However, cine-MRI is not precise due to a single imaging plane. To solve this issue, multiple imaging planes may be needed for a more realistic assessment of the 3D motion.

One method to estimate peristaltic motion may be acquiring sequential, high temporal resolution 3D volumes with the patient under breath hold. In this case, the respiration motion would be minimized, permitting estimation of peristaltic motion. Although residual motion may be present due to breath hold inconsistency, this could be rectified through image registration.

Peristaltic motion was found to be different between patients due to patient specific peristaltic filling. For instance, the amount of gas accumulated inside the stomach varied between patients. It was also observed that the amount of gas inside the stomach and bowels changed during the day.

The peristaltic motion, in addition to respiratory motion, should be properly estimated and managed in the precise treatment for abdominal tumor target. From Table 4, the average peristalsis motion of the pancreatic head and duodenum were measured from the absolute motion, which removes the negative and positive signs representing the directions of motions. Accordingly, the duodenum motion relative to pancreatic head motion shows a slight variation with duodenum and pancreatic head motion individually. In order to correct the variation and introduce a margin for duodenum and pancreatic head motion due to peristalsis, quadratic propagated uncertainty of the motions was selected. Therefore, an additional margin of 5 mm was found due to peristalsis motion, which needs to be added to planning target volume (PTV). For example, PTV-margin for peristaltic motion should be considered in the treatment planning and delivery of dose-escalated or hypofractionated RT/ SBRT for pancreatic cancer.

A weakness in the current study is that the center of mass motion of an organ was used for the motion assessment. However, for the organ that has deformation during respiration cycle, the center of mass motion may not completely characterize the whole motion of the organ. The study by Sheng et al showed that the center of mass motion of an organ with a small volume can represent its overall motion [24]. Furthermore, Xie et al and Van Sornsen de Koste et al demonstrated that center of mass motion can be used to estimate the organ motion even for organs with a large volume, but small deformation like prostate and lung lesions [25, 26]. These studies indicate that the center of mass motion can approximately represent the motion of some organs in abdomen that have either small volumes or small deformations (e.g., head of pancreas and duodenum), while it may not well describe the motion of an organ experiencing large deformation (e.g., stomach and bowels). It is seen from Fig 5B in the present study that the deformation of stomach causes a shift of stomach center of mass in different respiratory phases. In such a situation with a large deformation, segmenting the organ as a function of time and, then, creating a union of all contours obtained will lead to more accurate estimate of the organ motion than the center of mass motion. The accurate assessment of the organ motion is necessary to effectively manage the motion during radiation therapy.

## Conclusion

The complexity of abdominal organ motion due to peristalsis in the time frames from seconds to minutes was investigated in this study using various types of MRI and CT data. Peristaltic motion was found to be non-periodic with non-predictable pattern for each patient, can be large and can be different between different organs (e.g., duodenum vs. pancreatic head). The peristaltic motion should be considered together with respiratory motion during RT for abdominal tumors.

## Supporting information

S1 Video Motions of various organs including gallbladder (GB) and liver with Cine-MRI. (MP4)
